# RNA-Based Vaccines in Cancer Immunotherapy

**DOI:** 10.1155/2015/794528

**Published:** 2015-11-19

**Authors:** Megan A. McNamara, Smita K. Nair, Eda K. Holl

**Affiliations:** ^1^Department of Medicine, Duke University Medical Center, Durham, NC 27710, USA; ^2^Department of Surgery, Duke University Medical Center, Durham, NC 27710, USA; ^3^Department of Pathology, Duke University Medical Center, Durham, NC 27710, USA

## Abstract

RNA vaccines traditionally consist of messenger RNA synthesized by* in vitro* transcription using a bacteriophage RNA polymerase and template DNA that encodes the antigen(s) of interest. Once administered and internalized by host cells, the mRNA transcripts are translated directly in the cytoplasm and then the resulting antigens are presented to antigen presenting cells to stimulate an immune response. Alternatively, dendritic cells can be loaded with either tumor associated antigen mRNA or total tumor RNA and delivered to the host to elicit a specific immune response. In this review, we will explain why RNA vaccines represent an attractive platform for cancer immunotherapy, discuss modifications to RNA structure that have been developed to optimize mRNA vaccine stability and translational efficiency, and describe strategies for nonviral delivery of mRNA vaccines, highlighting key preclinical and clinical data related to cancer immunotherapy.

## 1. Introduction

Cancer immunotherapy seeks to stimulate a host antitumor immune response, leading to tumor shrinkage and improved clinical outcomes in patients. In recent years, this field has exploded with the development of many different classes of agents aimed at enhancing immune responses against tumors. These include cytokines, immune checkpoint inhibitors, adoptive T cell therapies, and numerous vaccine strategies [1–3]. Several of these new immunotherapies, particularly the immune checkpoint inhibitors, including ipilimumab in metastatic melanoma [4] and nivolumab in non-small cell lung cancer [5], have demonstrated impressive survival benefits in large phase III trials, leading to the FDA approval of these agents and solidifying immunotherapy as a new modality for the treatment of cancer.

Compared to some other types of cancer immunotherapy, vaccines can be more cumbersome to produce and, for the most part, have shown more modest clinical responses in patients [6–12]. However, they remain an attractive cancer treatment approach because they represent a specific, safe, and well-tolerated therapy that also offers the potential to avoid drug resistance and obtain durable treatment responses due to immunologic memory. There are four main categories of cancer vaccines: (1) peptide vaccines, (2) cellular vaccines, including tumor cell and immune cell vaccines, (3) viral vector vaccines, and (4) nucleic acid vaccines, including DNA and RNA vaccines. This review will begin with a brief overview of nucleic acid vaccines for context and will then focus on RNA cancer vaccines. We will provide a discussion of RNA structure, as it relates to RNA vaccine therapy. We will then describe key preclinical and clinical data for the different types of RNA cancer vaccines, highlight advantages and disadvantages of various methods of RNA vaccine delivery, and discuss potential combinations of RNA vaccines with other therapies.

## 2. Nucleic Acid Vaccines: Definition, Appeal of the Platform, and Advantages of RNA over DNA

By definition, nucleic acid vaccines are vaccines containing antigens encoded by either DNA or RNA. More specifically, DNA vaccines consist of antigen-encoding gene(s) inserted into a bacterial plasmid under the control of a eukaryotic promoter. The DNA plasmid is administered to the host and internalized by host cells, where it is transcribed in the nucleus and translated in the cytoplasm by host cellular machinery. The resulting proteins are processed into peptides, which are ultimately presented on the surface of host antigen-presenting cells (APC) in the context of major histocompatibility complex (MHC) molecules. This can occur by APC being directly transfected with the DNA or by cross-presentation from non-APC to APC. The peptide-MHC complex is recognized by antigen-specific T cells, resulting in a cellular host immune response [13]. Alternatively, RNA vaccines involve messenger RNA (mRNA) synthesized by* in vitro* transcription (IVT) using a bacteriophage RNA polymerase and template DNA that encodes the antigen(s) of interest. Once administered and internalized by host cells, the mRNA transcripts are translated directly in the cytoplasm and then, like DNA vaccines, the resulting antigens are presented to APC to stimulate an immune response [14]. It is important to recognize that mRNA-encoded products are degraded by proteasomes and presented on MHC class I molecules to CD8+ T cells and do not reach the MHC class II processing pathway to induce CD4+ T helper cell responses. However, several studies have demonstrated that addition of a lysosomal targeting signal to the antigen-encoding sequence can result in a productive T helper cell response [15, 16]. Additionally, tumor antigen mRNAs fused to a signal peptide and an HLA class II sorting can result in HLA class I and II presentation [17].

The nucleic acid vaccine platform is appealing because it allows easy delivery of multiple antigens with one immunization and induces both humoral and cellular immune responses, which makes tumor escape less likely. Additionally, unlike peptide-based vaccines, nucleic acid-based vaccines do not require prior knowledge and are not restricted by the patient's HLA type. Finally, like other vaccine types, nucleic acid-based vaccines have proven to be safe and tolerable [14, 18–20].

Despite the promising features of DNA vaccines, in general, they have been found to elicit less of an immune response than other types of vaccines, including peptide vaccines, cellular vaccines, viral vector vaccines, and RNA vaccines. The reasons for this are not completely clear, but possible explanations include inefficient delivery of DNA into human cells, the need for DNA to cross both cell and nuclear membranes and be transcribed in the nucleus in order to transfect a cell, low expression of DNA-sensing machinery, and differing expression of nucleic acid sensing pattern recognition receptors [11, 21]. The relatively poor immunogenicity of DNA vaccines combined with concerns about their potential for oncogenesis via integration into the host genome has driven a shift away from DNA vaccines and towards RNA vaccines.

RNA vaccines are attractive because they retain the same appealing characteristics as DNA vaccines but also offer some additional benefits. Unlike DNA, RNA only needs to gain entry into the cytoplasm, where translation occurs, in order to transfect a cell. Moreover, RNA cannot integrate into the genome and therefore has no oncogenic potential. In addition to* in vitro* transcription, RNA can also be isolated from a limited tumor sample and amplified using techniques such as polymerase chain reaction (PCR), yielding large amounts of patient-specific antigens [11, 14]. Finally, RNA can act as an adjuvant by providing costimulatory signals, for example, via toll-like receptors TLR3, TLR7, and TLR8 [22]. For these reasons, there is a growing interest in the research and development of RNA vaccines.

## 3. RNA Structure: 5′ Cap, Poly(A) Tail, UTR, and Chemically Modified Nucleosides

Eukaryotic mRNA is composed of a coding region flanked by 5′ and 3′ untranslated regions (UTR), as well as a 5′ 7-methylguanosine triphosphate (m^7^G) cap and a 3′ poly(A) tail. The m^7^G cap, poly(A) tail, and UTR are all critical for mRNA stability and translation [23–25]. A keen awareness of this mRNA biology is vital when developing RNA vaccines since mRNA stability and translational efficiency dictate the amount of antigen produced, which impacts the degree of immune response generated.

*5*′* m* ^*7*^ *G Cap*. A 7-methylguanosine triphosphate (m^7^G) cap is added to the 5′ end of almost all eukaryotic mRNA transcripts during transcription. The cap protects the 5′ end of the mRNA transcript from 5′ to 3′ exonucleases [26] and is recognized by the eukaryotic translation initiation factor eIF4E [27], thus playing a key role in both mRNA stability and translation.

Capping of IVT mRNA transcripts can be achieved using cap analogues. However, it has been shown that cap analogues are often incorporated in the reverse orientation with the methylated G proximal to the RNA [28], resulting in an inability to translate a substantial number of mRNA transcripts [29]. To address this issue, antireverse cap analogues (ARCA) have been designed that cannot be incorporated in the reverse orientation because they contain only one 3′-OH group, rather than the two 3′-OH groups contained on the initial cap analogues. Importantly, the translational efficiency of ARCA-capped mRNA transcripts is more than twice that of mRNA capped with conventional cap analogues [29]. Additionally, a higher level of protein expression, maintained for a longer duration of time, is achieved in cells transfected with ARCA-capped IVT mRNA transcripts, compared to cells transfected with mRNA transcripts capped with regular cap analogues [30].

Adding a cap analogue, conventional or ARCA, during IVT is not 100% efficient; thus a portion of the resulting transcripts is not capped at all. These uncapped RNAs are not efficiently translated. A method of adding a cap structure posttranscriptionally is gaining favorability among those developing RNA-based therapies.

*Poly(A) Tail*. A poly(A) tail is added to the 3′ end of the majority of eukaryotic mRNA transcripts during transcription. The poly(A) tail regulates mRNA stability and translation synergistically with the m^7^G cap by binding poly(A) binding protein (PABP) [31], which interacts with eukaryotic translation initiation factor eIF4G, which in turn forms a complex with the m^7^G cap and eIF4E [32].

There are two ways to add a poly(A) tail to IVT mRNA: (1) encoding the poly(A) tail on the DNA template from which the IVT mRNA is transcribed or (2) using recombinant poly(A) polymerase to extend the IVT mRNA after transcription. In contrast to enzymatic polyadenylation with recombinant poly(A) polymerase, which yields mRNA transcripts with poly(A) tails of varying lengths, mRNA transcribed from a DNA template yields transcripts with a defined poly(A) tail length and is therefore preferred [33]. Studies have shown that increasing the length of the poly(A) tail increases the efficiency of polysome formation [34] as well as the level of protein expression. The optimal length of the poly(A) tail in IVT mRNA appears to be between 120 and 150 nucleotides [33, 35, 36].

*5*′* and 3*′* Untranslated Regions*. Eukaryotic mRNA transcripts include 5′ and 3′ untranslated regions (UTR), which contain important regulatory elements.

IVT mRNA can be optimized by incorporating 5′ and 3′ UTR known to enhance RNA stability and translational efficiency. The most well recognized examples of such UTR in IVT mRNA are the alpha- and beta-globin mRNAs. Beta-globin 5′ and 3′ UTR improve translational efficiency, and alpha-globin 3′ UTR stabilize mRNA [37–39]. These globin UTR are used in many preclinical and clinical studies involving IVT mRNA [40–42].

*Chemically Modified Nucleosides*. Finally, IVT mRNA can be created by incorporating chemically modified nucleosides, which are known to reduce immunogenicity. Natural nucleosides are added to mammalian RNA during posttranslational RNA processing in eukaryotes [43]. This has been explored as a potential method to render IVT mRNA less immunogenic.

IVT mRNA containing modified nucleosides such as pseudouridine possesses increased stability and translation [44, 45]. This is thought to occur as a result of nucleosides rendering IVT mRNA undetectable by cytoplasmic TLRs such as TLR3, TLR7, and TLR8, as well as RIG-I and PKR [43, 44, 46]. Moreover, further processing of IVT mRNA by high-performance liquid chromatography purification to remove dsRNA contaminants results in reduced type I interferons and proinflammatory cytokines production [47], thus leading to increased and prolonged mRNA translation.

## 4. Nonviral Strategies for Delivery of mRNA Vaccines

The previously described modifications to the 5′ m^7^G cap, poly(A) tail, 5′ and 3′ UTR, and nucleosides are fundamental to optimize the stability and translational efficiency of all IVT mRNA for all RNA vaccines. Once an IVT mRNA transcript has been generated, it must be administered and ultimately must reach the cytoplasm of target cells. In general, nonviral delivery methods are preferred over viral vectors for their low cost, ease of large-scale production, and potential for improved safety [48, 49]. We will now review different nonviral strategies for delivery of mRNA vaccines, with a focus on their role in cancer immunotherapy.

### 4.1. “Naked” mRNA Vaccines

The initial data for the use of mRNA as a vaccine platform emerged 25 years ago when Wolff et al. demonstrated that intramuscular injection of mRNA coding for reporter genes induced* in vivo* expression of those reporter genes in mice [50]. Five years later, Conry et al. developed the first mRNA cancer vaccine by showing that mice immunized with mRNA coding for carcinoembryonic antigen (CEA) mounted an anti-CEA antibody response when challenged with CEA expressing tumor cells [51]. Both of these accomplishments represent examples of “naked” RNA vaccines, meaning the mRNA is injected directly, formulated only in buffer and without a carrier. Since then, numerous studies in animal models have confirmed that naked mRNA can transfect host cells and induce antigen-specific antibody and T cell immune responses [41, 52–56]. However, despite some encouraging early results, naked mRNA vaccines remain limited by the short extracellular half-life of naked mRNA due to rapid degradation by ubiquitous RNAases [57, 58]. Moreover, RNA vaccines induce transient protein expression, thus limiting the time for treatment effectiveness. This would in turn increase the number of times a patient visits the clinic for treatment. In addition to the previously described techniques to optimize the structure of IVT mRNA, different strategies, including two key techniques described below, have been tried in order to stabilize the naked mRNA, improve translational efficiency, and overcome this barrier.

*Gene Gun*. The gene gun is an alternative delivery method, in which IVT mRNA is injected directly into the target cell cytoplasm, thereby limiting exposure to extracellular exonucleases that might degrade it. More specifically, in the gene gun technique, IVT mRNA is coated onto gold particles, which are then accelerated toward a stopping plate by a pressurized helium pulse. The gold particles penetrate into the cytoplasm of target cells, serving as carriers for the mRNA [59, 60]. The gene gun has been shown to be an effective delivery mechanism for IVT mRNA in animal models. For example, gene gun-based immunization using IVT mRNA coding for the melanocyte self-antigen TRP2 linked to the immunogenic protein EGFP induced antigen-specific cellular and humoral immunity in mice and was protective against B16 melanoma lung metastases [53]. However, despite this success in animal models, the gene gun delivery method has not yet been translated into clinical trials in humans.

*Protamine Condensation*. Another method to improve the stability of naked IVT mRNA is to condense it in order to provide protection from RNA degradation. This can be accomplished by incubating IVT mRNA with protamine, a small arginine-rich polycationic protein, normally involved in DNA condensation [56]. Protamine condensation is also attractive because, in addition to stabilizing IVT mRNA, protamine acts as a danger signal and stimulates an immune response through MyD88, TLR7, and TLR8 dependent pathways [61–63]. Protamine-condensed IVT mRNA has been shown to induce specific cellular and humoral immune responses* in vivo* in both mice and humans. For example, the injection of protamine-protected naked mRNA into mice stimulated production of antigen-specific IgG antibodies as well as activation of a specific cytotoxic T lymphocyte response and effective lysis of target cells [56]. In a phase I/II clinical trial in 21 patients with metastatic melanoma, intradermal injection of protamine-condensed naked mRNA encoding six melanoma-associated antigens was feasible and safe, increased vaccine-directed T cells in two of the four evaluable patients, and yielded a complete response in one of the seven patients with measurable disease [64].

### 4.2. Adjuvants to mRNA-Based Vaccines

An adjuvant can be broadly defined as a component that is added to a vaccine to enhance the immunogenicity of the vaccine. Naked IVT mRNA possesses inherent self-adjuvanticity. Additionally, several other molecules, including protamine, as described previously, poly I:C RNA, and CpG containing motifs can be combined with naked IVT mRNA to augment an mRNA-based vaccine's ability to induce an adaptive immune response. However, even with the addition of an adjuvant, a vaccine may not be potent enough to overcome the powerful immunosuppressive effects of tumors. To address this issue, mRNA encoding costimulatory molecules, such as CD40L, CD70, OX40L, GITR, and CD83, can be incorporated into mRNA-based vaccines to further boost their immunogenicity [65–67].

### 4.3. Encapsulated mRNA Vaccines

Despite the previously described strategies to improve the stability of naked IVT mRNA, RNA degradation remains a significant concern with naked RNA vaccines. Because of this, carriers have been developed to encapsulate IVT mRNA, thereby protecting it from degradation and improving vaccine delivery [68]. To date, many such carriers have shown great potential in mRNA delivery to mammalian cells [69]. Cationic liposomes, specifically N-[1-(2,3-dioleoloxy)propyl]-N,N,N-trimethyl ammonium chloride 1(DOTAP), have been the most widely used encapsulating agents [69–72]. Despite these discoveries, the search continues for delivery agents that provide effective cytosolic mRNA delivery and are associated with limited* in vivo* cytotoxicity. Recent studies have shown that encapsulating mRNA in nanoparticles protects the mRNA from nuclease degradation and enhances cell uptake and delivery efficiency [73]. Moreover, these nanoparticles can be engineered to be fully degradable, a desired property in vaccine delivery. These particles contain a biodegradable core-shell structured nanoparticle with a pH responsive poly-(b-amino ester) (PBAE) core enveloped by a phospholipid shell [74]. Preclinical studies have shown that these particles are efficient in delivering mRNA* in vivo* and eliciting an antitumor immune response [66, 75, 76].

## 5. mRNA Transfected DC Vaccines

mRNA transfected dendritic cell (DC) vaccines represent a distinct type of vaccine strategy involving RNA. DCs are professional antigen presenting cells that play an essential role in bridging innate and adaptive immune responses. When used as a vaccination platform, dendritic cells (DCs) are transfected with mRNA encoding a desired tumor antigen and then delivered to the host in order to elicit an immune response against the antigen of interest. DCs can be transfected with tumor associated antigen (TAA) mRNA or total tumor RNA. Both methodologies have their advantages and drawbacks.

*Tumor Associated Antigens*. Vaccine strategies utilizing DCs transfected with defined TAA mRNA circumvent the need for growth of patient specific tumor cells and/or isolation of patient specific tumor antigen [77–79]. Moreover, the antigen preparation is homogenous and highly pure and the majority of the loaded DCs present the same epitope(s) on the surface. This vaccination strategy also lowers the risk of autoimmunity, which can be induced in patients by the inclusion of nonmutated, normally expressed endogenous proteins. However, TAA vaccination strategies come with many limitations. For many cancers the TAAs are not identified and significant investigation is still required. Vaccine development is costly and TAA selection can be difficult as not all identified TAAs elicit an antitumor immune response. Finally, when targeting a single antigen, there exists the possibility that the tumor itself will downregulate the TAA and allow escape.

Several studies to date have utilized TAA mRNA-loaded DCs to stimulated antitumor responses [80–84]. In a study by Heiser et al., DCs were transfected with prostate-specific antigen (PSA) TAA and administered into prostate cancer patients [85]. In this trial, the DC immunization elicited a PSA-specific T-cell response, which was accompanied by a significant decrease in PSA levels in six of seven patients. Additional studies have utilized CEA mRNA-loaded DCs to vaccinate patients with CEA expressing tumors [86, 87]. Although vaccination itself was well-tolerated, the antitumor response was limited to six (one complete response, two minor responses, and three stable diseases) out of 24 patients. Although we list many studies utilizing TAA mRNA vaccination strategies, this review cannot include all of the publications on this topic. Other articles in this issue will discuss additional vaccination outcome reports.

*Total Tumor RNA*. An alternative to the use of TAA to generate an anticancer immune response is the use of patient derived total tumor RNA [88–93]. This method utilizes cancer specific RNA and eliminates the need for identification of antigens expressed by the patient's tumor. Through this methodology the entire spectrum of tumor specific antigen is displayed, thus allowing the immune system to utilize the most effective antigens while reducing the risk of escape mutants. The advantage of using tumor-derived RNA as a source of whole-tumor antigen is that it can be quickly and easily amplified by RT-PCR from even a small amount of tumor. This allows for an unlimited supply of antigen, which differs from strategies using tumor lysate or cells [85].

Clinical studies utilizing total tumor RNA vaccination strategies have been tested in several tumor models including brain cancer, lung adenocarcinoma, melanoma, renal cell carcinoma, and ovarian cancer [94–100]. In the renal cell carcinoma study by Su et al., patients displayed no evidence of dose-limiting toxicity or induction of autoimmunity [100]. Similarly, brain tumor and neuroblastoma studies conducted in nine and seven patients, respectively, showed a clinical response in a total of three of the patients. Despite many advances in RNA-DC immunotherapies, clinical responses remain modest and new strategies on how to best prepare and administer these vaccinations are being explored.

Of note, loading DCs with total tumor RNA can lead to expression of self-proteins and induction of autoimmunity. However, this has not been an issue in the studies conducted thus far.

*Costimulatory Molecules and Checkpoint Inhibitors as Part of mRNA Transfected DC Vaccine Strategies*. The benefit of DC based vaccinations has been limited primarily due to the presence of regulatory T cells as well as upregulation of checkpoint molecules. Recent studies have focused on new strategies to enhance the efficacy of RNA transfected DC vaccines. Cotransfection of DCs with mRNA encoding OX40-ligand in addition to tumor antigen improves mRNA-DC vaccine efficacy in preclinical models [101]. Moreover, DCs activated through electroporation with tumor mRNA as well as mRNA encoding CD40 ligand and constitutively active TLR4 and CD70 (TriMix-DCs) are potent antigen presenting cells. They induce effector T cells that do not respond to regulatory T cell suppression, an important strategy to generate a broad and robust immune response [102, 103].

In addition, studies where expression of checkpoint inhibitors, such as PD-1, is silenced utilizing siRNA have shown improved vaccine efficacy [104, 105]. Other strategies have focused on increased DC function by cotransfecting tumor antigen mRNA with mRNA encoding for checkpoint molecules such as CTLA-4 and GITR [106]. These engineered DCs have the ability to not only present the tumor antigen of interest but also secrete anti-CTLA4 and anti-GITR, thus locally modulating immune checkpoints and the tumor microenvironment. Taken together these strategies have potential to prevent or reduce cancer recurrence.

### 5.1. Route of Vaccine Delivery

Antigen-specific immune responses have been achieved in animal models and in humans by administration of RNA vaccines via various routes, including intramuscular, intradermal, subcutaneous, intravenous, intrasplenic, intranodal, intratumoral, and intranasal methods. These strategies each have their own advantages and disadvantages. However, they have not all been compared directly, and therefore the optimal route of RNA vaccine delivery is not known. For a more detailed discussion of RNA vaccine routes of administration, please refer to the following review papers: intramuscular [50, 52]; intradermal [107]; subcutaneous [108]; intrasplenic [109] and intranodal [41].

## 6. Conclusion

The field of cancer immunotherapy has undergone many changes in recent years, and immunotherapy has now been clinically validated as an effective way to treat many types of malignancies [1–3]. RNA vaccines represent an attractive form of cancer immunotherapy because they enable delivery of large amounts of patient-specific antigens derived from a small tumor sample, are not HLA-restricted, induce humoral and cellular immune responses, provide costimulatory signals, have no oncogenic potential, and are well-tolerated. As discussed in this review, several techniques have been developed to improve IVT mRNA stability and translational efficiency and to optimize RNA vaccine delivery. Despite these advances, clinical responses to RNA vaccines remain modest.

Moving forward, combining RNA vaccines with other therapies that have distinct mechanisms of action could potentially result in better treatment outcomes. For example, the vaccination methods described above could be used simultaneously or sequentially with immune checkpoint inhibitors such as anti-CTLA-4, anti-PD-1, and anti-PD-L1 [110]. In such a combination strategy, RNA vaccines could prove important in specifically targeting tumors, reducing tumor burden, and causing tumor cell lysis and antigen spread, while immune checkpoint blockade could be essential in perpetuating the immune responses and leading to better tumor cell clearance.

Moreover, in this review we discuss the importance of RNA strategies to directly block immune checkpoints and engineer the tumor microenvironment, similarly to antibodies against immune checkpoints. These strategies would perhaps limit the toxicities and side effects associated with systemic delivery of antibodies against immune checkpoints.

Further studies are needed to specifically investigate combinations of RNA vaccines with other immunotherapies as well as targeted and cytotoxic agents, with the overall goal of improving clinical outcomes and cancer care.
